# Exposure to Heavy Metals in Electronic Waste Recycling in Thailand

**DOI:** 10.3390/ijerph17092996

**Published:** 2020-04-26

**Authors:** Chalermkhwan Kuntawee, Kraichat Tantrakarnapa, Yanin Limpanont, Saranath Lawpoolsri, Athit Phetrak, Rachaneekorn Mingkhwan, Suwalee Worakhunpiset

**Affiliations:** 1 Department of Social and Environmental Medicine, Faculty of Tropical Medicine, Mahidol University 420/6 Ratchawithi Rd., Ratchathewee, Bangkok 10400, Thailand; 2 Ubon Ratchathani Provincial Health Office, 257 Phromthep Rd., Mueang District, Ubon Ratchathani 34000, Thailand; 3 Department of Tropical Hygiene, Faculty of Tropical Medicine, Mahidol University 420/6 Ratchawithi Rd., Ratchathewee, Bangkok 10400, Thailand

**Keywords:** heavy metals, asthma, house dust, airborne dust, e-waste

## Abstract

Electronic waste recycling can release heavy metals into the environment and cause adverse health effects. We assessed the association between exposure to heavy metals from electronic waste recycling and the prevalence of asthma in a nested case-control study of 51 subject pairs with and without asthma. House dust, airborne dust, blood, and urine were collected from residents of two neighboring sites in Ubon Ratchathani province, Thailand. Multiple electronic waste-handling activities are conducted in the first site, while the second site is mostly agricultural. Concentrations of chromium, mercury, nickel, and lead in house dust and airborne dust were higher in the electronic waste-handling site (*p* < 0.05), but levels of exposure were similar in subjects with and without asthma. Although we did not find an association between exposure to these metals and the prevalence of asthma, control measures should be implemented to reduce health risks from long-term exposure to heavy metals.

## 1. Introduction

Electronic waste (e-waste), discarded electrical and electronic equipment that is outdated, damaged, or malfunctioning, is one of the major sources of environmental and health-related problems worldwide [1,2]. It has emerged as the fastest growing waste stream due to technological advancement, fashion style, market expansion, end of product life, and misuse or lack of maintenance of electrical and electronic equipment. Since e-waste contains several toxic substances, inappropriate management can contribute to environmental contamination leading to adverse effects on human health, the environment and ecological systems [3,4]. Especially, in the informal sector, manual dismantling and open burning is commonly used for recovering valuable materials without adequate and proper personal protective equipment (PPE), which results in significant risk of exposure to toxic substances of the workers and communities [5,6]. In addition, several studies have reported levels of toxic substances such as heavy metals and persistent halogenated compounds in air, water, soil, sediment, and dust in and around e-waste sites [7,8,9,10].

The potential adverse health consequences from exposure to toxic substances in e-waste vary by substance, concentration and duration of exposure [11]. For example, lead (Pb) can affect the liver kidney, and nervous system and disrupt cognitive development [12,13,14]. Chromium (VI) can cause respiratory tract irritation, kidney and liver damage, weakened immune systems, and nasal, sinus or lung cancer. [15]. Mercury (Hg) exposure may lead to memory loss, immune toxicity, and muscle weakness [16]. Meanwhile, nickel (Ni) might contribute to dermatitis and bronchial asthma [17]. In addition, persistent halogenated compounds such as brominated flame retardants can interfere with hormone function and cause neurobehavioral effects, behavioral disorders, and cancer [16,18,19]. E-waste workers are at risk from direct exposure to toxic substances during work, whereas others may be exposed to toxic substances contaminating the environment.

Asthma is a respiratory disorder caused by chronic inflammation of the bronchi that results in airway hyperresponsiveness to stimuli such as allergens or irritants. The symptoms of asthma include shortness of breath, wheezing, chest tightness, and cough [6,20]. A wide variety of natural and man-made materials can cause sensitization of the respiratory tract as chemical allergens, including heavy metals [21,22]. Additionally, dermal absorption may also represent a relevant route through which respiratory sensitization can be achieved [23]. Exposure to lead (Pb) and mercury (Hg) has been associated with asthma via increased IgE levels [24], and more than 50% of asthma cases are hypothesized to be mediated by elevated IgE, which may also influence the association between blood levels of heavy metals [25].

Similar to other countries, the e-waste problem in Thailand has become an increasing concern. In 2013, Thailand generated 0.56 million tons of hazardous waste from households, and approximately 65% was e-waste [7], much of which went to informal sectors such as pickers, waste buyers, junk shops, recyclers, and re-processors. Inappropriate collecting, dismantling, recycling, and disposing may contribute to detrimental effects to human health and the environment, especially for the workers who carry out e-waste related activities without protective equipment [6].

The BanKok sub-district of Kuengnai district, Ubon Ratchathani province, has been involved in e-waste activities since 2011. In 2015, the Bureau of Environmental Health determined that the overall levels of heavy metals were below recommended limits [26]. Exposure to heavy metals can lead to bronchial inflammation [27,28], but a direct association to asthma has not been shown conclusively. The prevalence of asthma in BanKok has been shown to be double that of an area with no e-waste activities [29,30], perhaps owing to heavy metal exposure from activities related to e-waste.

The present study assessed the association between heavy metal exposure and the prevalence of asthma by measuring metal levels in house dust, airborne dust, and biological samples from asthmatic and non-asthmatic individuals residing in an area with e-waste activities and in a reference area without e-waste activities. The findings may contribute to the formulation of strategies for preventing adverse health effects from heavy metals in e-waste.

## 2. Materials and Methods

### 2.1. Study Sites

**Figure 1:**
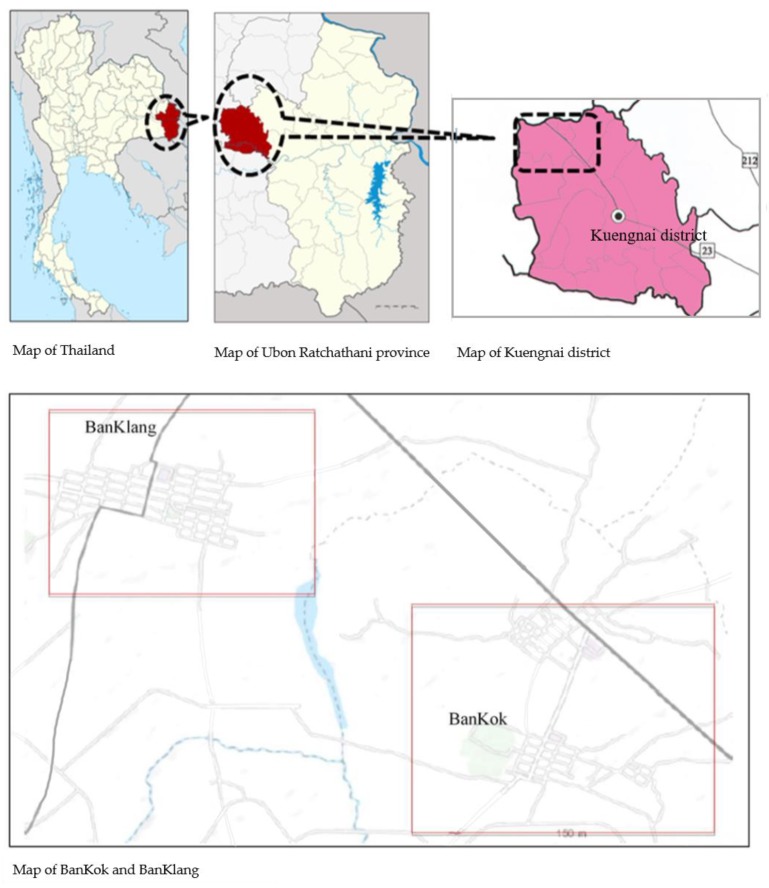
Map of the study sites.

The study sites were the BanKok and BanKlang sub-districts of Kuengnai district, Ubon Ratchathani province, northeastern Thailand, which are located approximately 590 km from Bangkok (Figure 1). BanKok is an e-waste recycling site and is estimated to include 23 businesses and 76 households engaged in e-waste processing. BanKlang, located 2 km from BanKok, is mostly agricultural and has no e-waste activities. 

### 2.2. Study Design and Population

We designed the investigation as a nested case-control study. The inclusion criteria were Thai nationality, age 20–70 years, resident of BanKok or BanKlang for more than 5 years, and no history of asthma (code J45 in the International Classification of Diseases, 10th revision) before 2011. A case was an asthma patient and a control was a non-asthmatic individual matched to the case by age and sex. All registered asthma cases in BanKok and BanKlang were included in this study. This study was approved by the Ethics Committee of the Faculty of Tropical Medicine, Mahidol University (approval MUTM 2017-008-01) and was conducted in accordance with the 1964 Helsinki declaration (and later amendments) or other comparable ethical standards. Written informed consent was obtained from each subject. 

### 2.3. Environmental and Biological Sample Collection and Analysis

#### 2.3.1. House Dust and Airborne Dust Collection and Analysis

House dust and airborne dust samples were collected from the residences of participants by purposive sampling between May and July 2017. House dust samples were collected from four sub-areas of 0.25 m^2^ for a total area of 1 m^2^ of the floor using dried and pre-weighed task wipes (Kimberly-Clark, Irving, TX, USA) and processed using the American Society for Testing and Materials (ASTM) method D6966-13 [31] with minor modifications. All subjects were asked to carry personal air samplers with a pre-weighted mixed cellulose ester membrane filter (0.8 µm pore size) that operated continuously for 8 h at a flow rate of 4 L/min to collect airborne dust in the daytime. The collected task wipes and membrane filters were kept in a resealable plastic bag, transported to the laboratory, placed in a desiccator for 24 h prior to weighing, and stored at −20 °C until analysis.

Task wipes and membrane filters were cut into small segments, digested in a microwave oven in a solution of pure concentrated nitric acid (65% HNO_3_), and filtered through a 0.45-µm Millipore filter paper (MilliporeSigma, Burlington, MA, USA) to obtain a clear solution. The filtrate was stored at 4 °C in polypropylene bottles for heavy metal analysis. 

#### 2.3.2. Blood and Urine Sample Collection 

Whole blood was obtained by venipuncture, collected in tubes with heparin as an anticoagulant, and stored at 4 °C until analysis. A spot urine sample was collected from the first morning urine and stored at −20 °C until analysis.

#### 2.3.3. Analysis of Heavy Metals

Concentrations of chromium (Cr), Hg, Ni, and Pb in dust samples were analyzed using inductively coupled plasma-optical emission spectrometry. Blood Pb and urinary Cr and Ni were analyzed using graphite furnace atomic absorption spectrophotometry (model Z-8200 polarized Zeeman atomic absorption spectrophotometer, Hitachi, Tokyo, Japan). Urinary Hg was determined using a mercury analyzer (NIC MA-3000, Nippon Instrument Corporation, Tokyo, Japan). A calibration curve was generated with known concentrations of each heavy metal from standard solutions. The solvents and chemicals used in this study were of analytical grade.

#### 2.3.4. Questionnaires

We conducted interviews with participants to collect information on socio-demographic and lifestyle factors that may be related to heavy metal exposure, as well as information on the use of personal protective equipment.

### 2.4. Data Analysis

Data obtained from this study were analyzed using SPSS version 19 (IBM SPSS, Armonk, NY, USA). After performing a Komogorov-Smirnov test, a Student’s *t*-test or the chi-squared test or the Mann-Whitney U test were used to compare general characteristics and heavy metal concentrations in the samples between asthma cases and controls. The level of significance was set at α = 0.05.

## 3. Results

### 3.1. General Characteristics of Study Subjects

A total of 102 subjects (84 from BanKok and 18 from BanKlang) participated in the study. There were 51 asthma cases (42 from BanKok and 9 from BanKlang). The control group consisted of 42 residents of BanKok and 9 residents of BanKlang. The majority of subjects (60) were female. Most participants were aged 47–68 years and had a primary school education and monthly income of 1000–5699 baht. Six asthma patients reported experiencing symptoms weekly, 12 experienced symptoms monthly, and 33 experienced symptoms less frequently, such as once every 6 months (Table 1).

### 3.2. Occupational and Environmental Factors Related to E-Waste Handling

**Figure 2:**
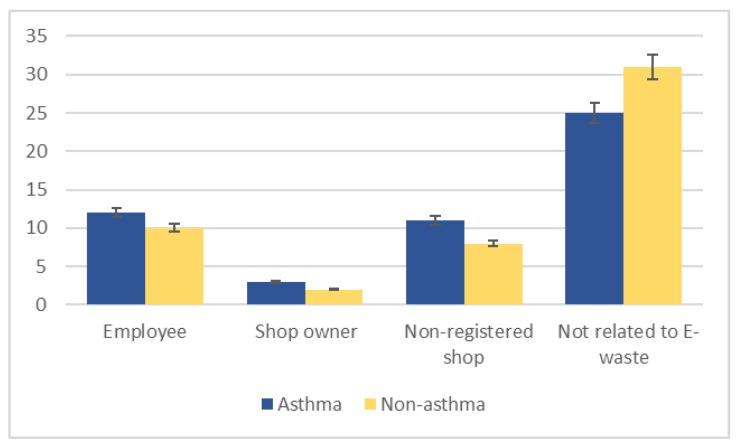
Participant involvement in electronic waste (e-waste) activities, stratified by asthma status.

**Figure 3:**
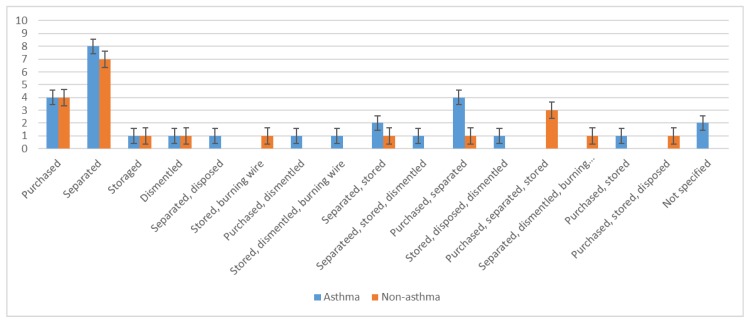
Participant involvement in specific steps of electronic waste handling, stratified by asthma status.

Twenty-six asthma cases were involved in e-waste activities (Figure 2). Of the asthma patients involved in e-waste activities, eight separated the waste, four were involved in purchasing it, another four both purchased and separated e-waste, and two separated and collected it; a few did not specify their involvement (Figure 3). Most worked ≥ 5 h per day, 6 days per week, and had been involved in e-waste activities for at least 5 years. Almost a third of all asthma patients lived near an e-waste shop, 23 had family members who worked with e-waste, and 10 cooked at an e-waste site.

Twenty non-asthmatic subjects were involved in e-waste activities. A third separated e-waste, four were involved in purchasing it, and others purchased or collected it (Figure 3). Similarly to the asthma cases, most worked ≥ 5 h per day, 6 days per week, and had been involved in e-waste activities for at least 5 years. Approximately a fourth of all non-asthmatic subjects resided near an e-waste shop, 13 had family members who were involved in e-waste activities, and only three cooked at an e-waste site. Fifty-six participants (25 asthma patients and 31 non-asthmatic subjects) were not involved in handling e-waste. 

**Figure 4:**
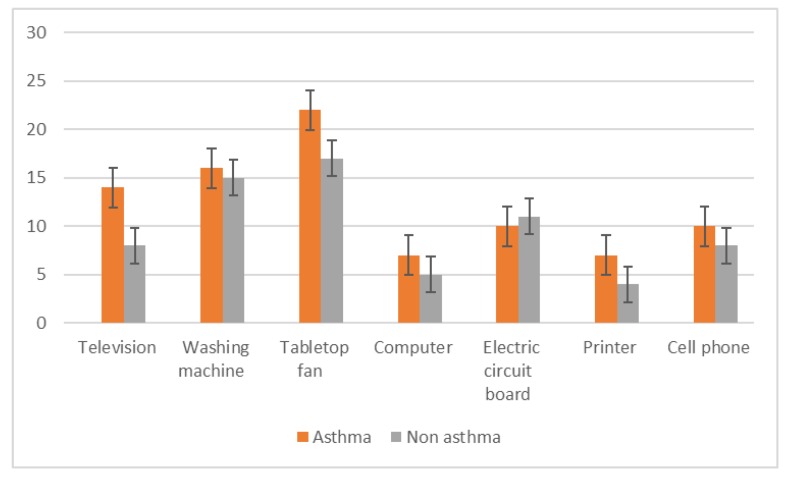
Types of electronic waste items handled by study participants.

**Figure 5:**
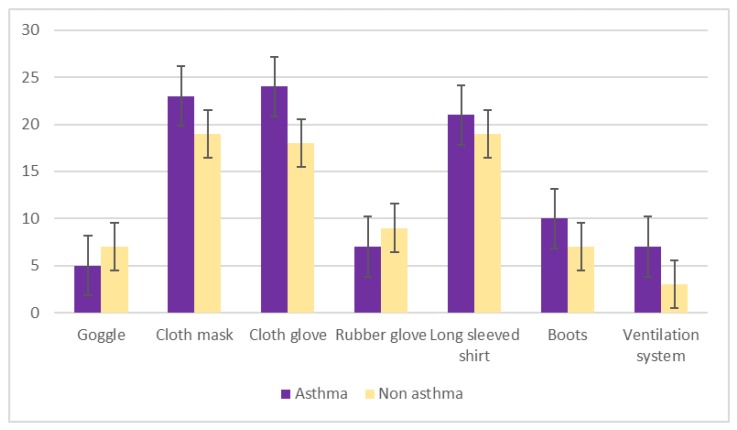
Use of personal protective equipment by study participants handling electronic waste.

The e-waste items most typically handled by study participants were tabletop fans, washing machines, televisions, and cell phones (Figure 4). Asthmatic subjects were more likely to use personal protective equipment when handling e-waste, including the facility’s ventilation system (Figure 5).

### 3.3. Heavy Metal Concentrations in House Dust and Airborne Dust of E-Waste and Non-E-Waste Related House

Average concentrations of Cr, Hg, Ni, and Pb in house dust and airborne dust samples collected from participants involved in e-waste activities were higher than concentrations in samples collected from participants who were not involved in e-waste activities (Table 2). 

### 3.4. Heavy Metal Concentrations in House Dust and Airborne Dust Stratified by Village

Average concentrations of Cr, Hg, Ni, and Pb in house dust and airborne dust were higher in BanKok than in BanKlang (*p* < 0.05) (Table 3). No difference in metal concentrations was observed between samples collected from asthmatics and non-asthmatics (*p* > 0.05), with the exception of Cr concentrations in airborne dust, which were higher in the breathing zone of asthmatics (*p* = 0.049) (Table 4). 

### 3.5. Heavy Metal. Levels in Blood and Urine

We measured the concentrations of metals in blood and urine as markers of exposure in participants. Mean concentrations of blood Pb and urinary Cr and Ni were slightly lower in the asthma group, but the difference did not reach statistical significance. Urinary Hg levels were higher in the asthma group (Table 5). 

Mean concentrations of urinary Cr, Hg, and Ni were higher in subjects who handled e-waste than in those who did not, but the difference did not reach statistical significance. Blood Pb levels were significantly higher in subjects who were not involved in e-waste activities (Table 6). No associations were found between use of personal protective equipment and blood Pb or urinary Cr, Hg, and Ni. 

Urinary Hg and Ni were higher in the BanKok group than in the BanKlang group, but blood Pb and urinary Cr were higher in the BanKlang group. However, only urinary Cr levels differed significantly between groups (Table 7). 

### 3.6. Association with Asthma

The chi-squared test found no significant differences between subject characteristics or occupational factors such as use of personal protective equipment and asthma (*p* > 0.05), with one exception: years of work were associated with a higher likelihood of asthma (*p* < 0.05) (Table 8).

## 4. Discussion

Associations between exposure to environmental and industrial toxicants and increased prevalence of asthma have been reported [32]. E-waste handling and recycling activities are considered a source of heavy metals whose adverse effects may include respiratory and other conditions [33,34]. We assessed the association between environmental exposure to several metals and the prevalence of asthma in sites with and without e-waste activities. However, some participants from BanKlang, the reference site, disclosed their involvement with e-waste. We therefore analyzed e-waste-related activities at the participant level. 

### 4.1. Heavy Metal Concentrations in Air and Dust

House dust and airborne levels of Cr, Hg, Ni, and Pb were higher in the homes and environment of subjects involved in e-waste activities. This is in accordance with the findings of several studies [10,35,36,37,38]. Metal concentrations did not vary significantly between house dust and airborne dust, perhaps because participants were not involved in activities that released airborne particulate matter. 

We also found that the metal concentrations were higher in BanKok, the e-waste site, than in BanKlang, the reference area without e-waste recycling facilities. The overall concentrations of Cr, Hg, Ni, and Pb measured in this study were lower than those reported previously in China and Nigeria [39,40,41], probably because our study subjects were mostly collectors and separators of e-waste and not involved in dismantling electronic devices. 

### 4.2. Heavy Metal. Concentrations in Blood and Urine

Higher median concentrations of blood Pb and Cr and urinary Ni have been reported in e-waste workers in Ghana [42], but we did not find significantly higher concentrations of metals in the blood and urine of e-waste workers. In contrast, we observed higher urinary Cr levels in subjects who were not involved in e-waste activities. 

Moreover, we did not observe a connection between exposure to heavy metals and asthma in our cohort. This may be attributable to the low levels of heavy metals we recorded, perhaps because little dismantling of electronic devices was conducted, leading to low levels of emissions. The high humidity in ambient air during the sample collection period may have also caused dust to settle rather than remain airborne. In addition, economic downturns in the US reduced global demand for metal in 2015, leading to lower market prices and decreased e-waste recycling in Thailand [43].

A recent case-control study in a Chinese cohort of 551 asthma patients and matched controls reported that asthma prevalence in adults was positively associated with urinary levels of some metals, including Cr, and negatively associated with levels of Ni and Pb [44]. Blood Pb levels did not differ between participants from BanKok and BanKlang, suggesting either that the e-waste in BanKok did not contain appreciable levels of Pb or that the residents of BanKlang were exposed to similar levels of Pb from agricultural fertilizers.

### 4.3. Association with Asthma

We did not find an association between blood or urinary concentrations of metals and asthma, perhaps because our cohort was small and metal levels were low.

## 5. Conclusions

We measured higher concentrations of metals in the house dust and breathing zone of subjects involved in handling e-waste than in those of subjects who did not engage in e-waste activities, but blood and urinary metal levels did not follow this trend. We did not find an association between exposure to heavy metals and the prevalence of asthma, probably because of the small sample, but control measures should be implemented to reduce health risks from long-term exposure to heavy metals. In addition, the association between the exposure to heavy metals and other toxic substances such as flame retardants from e-waste and asthma prevalence in a larger population-based cohort should be carried out.

## Figures and Tables

**Table 1:** Demographic information of participants stratified by site and group.

Demographic Characteristic	BanKok		BanKlang		Total	
	Asthma (n = 42)	Non-Asthma (n = 42)	Asthma (n = 9)	Non- Asthma (n = 9)	Asthma (n = 51)	Non- Asthma (n = 51)
Age (years)						
24–46	14	14	1	0	15	14
47–68	25	25	7	8	32	33
≥ 68	3	3	1	1	4	4
Mean ± SD	51.5 ± 12.1	51.5 ± 11.3	58.8 ± 6.8	57.8 ± 8.6	52.8 ± 11.6	52.8 ± 11.0
Gender						
Male	17	18	4	3	21	21
Female	25	24	5	6	30	30
Education						
Primary	34	33	6	7	40	40
Secondary	1	5	1	1	2	6
High school	7	4	1	1	8	5
Income (unit: Thai Baht)						
1000–5699	22	31	6	5	28	36
5700–10,399	18	11	3	3	21	14
≥ 10,400	7	0	0	0	7	0
Mean ± SD	6095.2 ± 3621.1	4542.9 ± 2474.6	4433.3 ± 3651.0	6444.4 ± 4186.6	5801.9 ± 3646.2	4878.4 ± 2891.7
Smoking						
None	33	30	7	5	40	35
Ex-smoke	1	2	1	2	2	4
Smoker	8	10	1	2	9	12
Frequency of asthma symptom						
1 time per week	4	-	2	-	6	-
1 time per month	10	-	2	-	12	-
1 time per six months	28	-	5	-	33	-

**Table 2:** Heavy metal concentrations in house dust and airborne dust collected from study participants.

	E-Waste Related House (46)			Non-E-Waste Related House (56)			*p*
	Range	Mean ± SD	Median IQR	Range	Mean ± SD	Median IQR	
House dust (mg/kg)							
Cr	ND–6.33	1.69 ± 1.29	1.52	0.02–5.87	0.99 ± 1.05	0.94	0.000
Hg	ND–1.04	0.09 ± 0.20	0.10	ND–0.79	0.04 ± 0.11	0.04	0.077
Ni	ND–7.88	1.46 ± 1.42	1.23	ND–12.00	0.92 ± 1.61	0.79	0.005
Pb	ND–79.27	7.53 ± 13.46	6.94	ND–16.93	3.00 ± 3.86	0.94	0.001
Air dust (µg/m^3^)							
Cr	ND–0.13	0.03 ± 0.03	0.03	ND–0.14	0.03 ± 0.04	0.05	0.318
Hg	ND–0.25	0.14 ± 0.06	0.09	ND–0.26	0.11 ± 0.07	0.12	0.013
Ni	ND–0.11	0.03 ± 0.03	0.05	ND–0.13	0.03 ± 0.03	0.05	0.842
Pb	ND–1.66	0.09 ± 0.25	0.11	ND–0.23	0.06 ± 0.07	0.12	0.620

IQR, interquartile range; ND, not detected; SD, standard deviation. Mann-Whitney U test significance set at α = 0.05.

**Table 3:** Average concentrations of chromium (Cr), mercury (Hg), nickel (Ni), and lead (Pb) in house dust and airborne dust stratified by village.

	BanKok (84)			BanKlang (18)			*p*
	Range	Mean ± SD	Median IQR	Range	Mean ± SD	Median IQR	
House dust (mg/kg)							
Cr	ND–6.33	1.51 ± 1.24	1.56	0.06–1.11	0.37 ± 0.30	0.40	0.000
Hg	ND–1.04	0.07 ± 0.17	0.08	ND–0.10	0.02 ± 0.03	0.04	0.328
Ni	ND–12.00	1.32 ± 1.65	1.11	ND–1.55	0.45 ± 0.42	0.51	0.000
Pb	ND–79.27	5.86 ± 10.49	6.49	ND–5.43	1.24 ± 1.66	2.09	0.000
Air dust (µg/m^3^)							
Cr	ND–0.14	0.03 ± 0.04	0.05	ND–0.06	0.01 ± 0.02	0.01	0.001
Hg	ND–0.26	0.14 ± 0.06	0.09	ND–0.12	0.05 ± 0.03	0.02	0.000
Ni	ND–0.13	0.04 ± 0.04	0.06	ND	ND	ND	0.000
Pb	ND–1.66	0.09 ± 0.19	1.24	ND–0.12	0.01 ± 0.03	ND	0.000

IQR, interquartile range; ND, not detected; SD, standard deviation. Mann-Whitney U test significance set at α = 0.05.

**Table 4:** Average concentrations of chromium (Cr), mercury (Hg), nickel (Ni), and lead (Pb) in house dust and airborne dust stratified by asthma status.

	Asthma (51)			Non Asthma (51)			*p*
	Range	Mean ± SD	Median IQR	Range	Mean ± SD	Median IQR	
House dust (mg/kg)							
Cr	ND–6.33	1.31 ± 1.23	1.35	0.03–5.87	1.30 ± 1.21	1.48	0.894
Hg	ND–0.79	0.06 ± 0.16	0.07	ND–1.04	0.07 ± 0.16	0.08	0.944
Ni	ND–4.60	1.02 ± 0.87	0.87	ND–12.00	1.31 ± 1.99	1.41	0.671
Pb	ND–23.47	3.83 ± 4.64	4.64	ND–79.27	6.26 ± 12.76	6.00	0.955
Air dust (µg/m^3^)							
Cr	ND–0.14	0.03 ± 0.03	0.05	ND–0.13	0.02 ± 0.03	0.04	0.049
Hg	0.02–0.25	0.13 ± 0.07	0.13	ND–0.26	0.12 ± 0.07	0.08	0.651
Ni	ND–0.13	0.03 ± 0.03	0.06	ND–0.13	0.03 ± 0.03	0.05	0.294
Pb	ND–0.23	0.07 ± 0.07	0.12	ND–1.66	0.08 ± 0.23	0.12	0.629

IQR, interquartile range; ND, not detected; SD, standard deviation. Mann-Whitney U test significance set at α = 0.05.

**Table 5:** Average concentrations of blood lead (Pb) and urinary mercury (Hg), nickel (Ni), and chromium (Cr) in study subjects stratified by asthma status.

Heavy Metal in Biological Sample Heading	Asthma (51)			Non Asthma (51)			*p*
	Range	Mean ± SD	Median IQR	Range	Mean ± SD	Median IQR	
Blood Pb (µg/dL)	0.89–8.50	4.80± 1.97	3.14	1.61–9.67	4.83 ± 2.16	3.23	0.857 *
Urinary Cr (µg/g creatinine)	ND–4.99	0.69 ± 0.99	0.53	0.06–5.03	0.71 ± 0.89	0.64	0.410 **
Urinary Hg (µg/g creatinine)	0.07–52.64	3.69± 8.60	1.51	0.31–24.16	3.06 ± 4.43	1.81	0.933 **
Urinary Ni (µg/g creatinine)	0.73–29.82	5.63 ± 6.66	3.33	0.25–99.96	6.81 ± 14.44	3.76	0.960 **

IQR, interquartile range; ND, not detected; SD, standard deviation. * *t*-test; ** Mann-Whitney U test set at α = 0.05.

**Table 6:** Average concentrations of blood lead (Pb) and urinary mercury (Hg), nickel (Ni), and chromium (Cr) in study subjects stratified by involvement in electronic waste (e-waste) activities.

Heavy Metal in Biological Sample	E-Waste (46)			Non E-Waste (56)			*p*
	Range	Mean ± SD	Median IQR	Range	Mean ± SD	Median IQR	
Blood Pb (µg/dL)	0.89–9.67	4.69 ± 2.16	3.24	1.94–9.41	4.91± 1.90	3.11	0.749 *
Urinary Cr (µg/g creatinine)	0.02–5.03	0.71 ±1.05	0.52	0.06–4.51	0.69 ± 0.84	0.59	0.574 **
Urinary Hg (µg/g creatinine)	0.07–52.64	4.64 ± 9.36	2.29	0.25–24.16	2.34 ± 3.35	1.04	0.382 *
Urinary Ni (µg/g creatinine)	0.44–99.96	8.34 ± 15.87	4.99	0.25–23.54	4.48 ± 4.53	2.91	0.352 **

IQR, interquartile range; ND, not detected; SD, standard deviation. * *t*-test; ** Mann-Whitney U test set at α = 0.05.

**Table 7:** Average concentrations of blood lead (Pb) and urinary mercury (Hg), nickel (Ni), and chromium (Cr) in study subjects stratified by location.

Heavy Metal in Biological Sample	BanKok (84)			BanKlang (18)			*p*
	Range	Mean ± SD	Median IQR	Range	Mean ± SD	Median IQR	
Blood Pb (µg/dL)	0.89–9.67	4.70 ± 2.11	3.27	2.74–7.98	5.34± 1.39	1.68	0.608 *
Urinary Cr (µg/g creatinine)	ND–5.03	0.68 ±0.99	0.49	0.15–2.54	0.80 ± 0.64	0.75	0.040 **
Urinary Hg (µg/g creatinine)	0.07–52.64	3.78 ± 7.45	1.86	0.33–3.36	1.50 ± 0.73	1.05	0.2260 **
Urinary Ni (µg/g creatinine)	0.30–99.96	6.48 ± 12.16	3.57	0.25–23.54	4.99 ± 5.55	4.47	0.629 **

IQR, interquartile range; ND, not detected; SD, standard deviation. * *t*-test; ** Mann-Whitney U test set at α = 0.05.

**Table 8:** Association of personal and occupational characteristics and asthma in study participants.

	Variables	Dependent Variables		*p*-Value
		Asthma (%)	Non Asthma (%)	
Subject characteristics				
Age group (years)				
	24–46.99	14	16	0.907
	47–68.99	33	31	
	≥69	4	4	
Income (Thai baht)				
	1000–5699	28	35	0.236
	5700–10,399	22	14	
	≥10,400	1	2	
Education				
	Primary	41	39	0.405
	Higher than primary	40	12	
Smoking behavior				
	None smoker	40	37	0.730
	Ex-smoker	2	4	
	Smoker	9	10	
Occupational factors				
Current work				
	Related to E-waste	26	20	0.233
	Not related to E-waste	25	31	
Position in E-waste activity				
	Employee	12	10	0.683
	Registered owner	3	2	
	Non registered owner	11	8	
	Not related to E-waste	25	31	
Work hours (Hours per day)				
	1–2.99	8	11	0.736
	2–5.99	18	16	
	≥6	25	24	
Years of work				
	1–2.99	30	31	0.035
	3–5.99	7	12	
	≥6	14	8	
Family member works in e-waste activity				
	yes	22	13	0.061
	no	29	38	
House beside e-waste shop				
	yes	16	12	0.375
	no	35	35	
House cleaning				
	7 days per week	26	24	0.912
	2–3 days per week	18	20	
	1 day per week	7	7	
Kitchen in E-waste related activities area				
	yes	9	3	0.065
	no	42	48	
Blood lead level (µg/dL)				
	0.89–5.29	28	31	0.574
	≥5.30	23	20	
Urinary chromium (µg/g creatinine)				
	0.06–2.59	49	49	1.000
	≥2.60	2	2	
Urinary nickel (µg/g creatinine)				
	0.30–33.39	51	49	0.361
	33.40–66.49	0	1	
	≥66.50	0	1	
Urinary mercury (µg/g creatinine)				
	0.07–29.99	49	51	0.153
	≥30	2	0	
